# Cord blood cardiovascular biomarkers in tetralogy of fallot and D-transposition of great arteries

**DOI:** 10.3389/fped.2023.1151814

**Published:** 2023-04-28

**Authors:** Olga Gómez, Laura Nogué, Iris Soveral, Laura Guirado, Nora Izquierdo, Miriam Pérez-Cruz, Narcís Masoller, María Clara Escobar, Joan Sanchez-de-Toledo, Josep Maria Martínez-Crespo, Mar Bennasar, Fàtima Crispi

**Affiliations:** ^1^ BCNatal Fetal Medicine Research Center, Sant Joan de Déu Hospital, Barcelona, Spain; ^2^ August Pi i Sunyer Biomedical Research Institute (IDIBAPS), Barcelona, Spain; ^3^ Fetal Medicine Department, Centro de Investigación Biomédica en Red de Enfermedades Raras (CIBERER), Madrid, Spain; ^4^ Department of Obstetrics, Hospital General de Hospitalet, Barcelona, Spain; ^5^ Sant Joan de Déu Research Institute (IRSJD), Barcelona, Spain; ^6^ Primary Care Interventions to Prevent Maternal and Child Chronic Diseases of Perinatal and Developmental Origin Network, Carlos III Health Institute, Madrid, Spain; ^7^ Pediatric Cardiology Department, Sant Joan de Déu Hospital, Esplugues de Llobregat, Barcelona, Spain

**Keywords:** transforming growth factor beta, Troponin I, angiogenic factors, congenital heart disease, fetal echocardiography, NT-pro-brain natriuretic peptide, tetralogy of fallot, transposition of the great arteries

## Abstract

Previous reports suggest that cord blood biomarkers could serve as a prognostic tool for conotruncal congenital heart defects (CHD). We aimed to describe the cord blood profile of different cardiovascular biomarkers in a prospective series of fetuses with tetralogy of Fallot (ToF) and D-transposition of great arteries (D-TGA) and to explore their correlation with fetal echocardiography and perinatal outcome.

**Methods:**

A prospective cohort study (2014–2019), including fetuses with isolated ToF and D-TGA and healthy controls, was conducted at two tertiary referral centers for CHD in Barcelona. Obstetric ultrasound and fetal echocardiography were performed in the third trimester and cord blood was obtained at delivery. Cord blood concentrations of N-terminal precursor of B-type natriuretic peptide, Troponin I, transforming growth factorβ (TGFβ), placental growth factor, and soluble fms-like tyrosine kinase-1 were determined.

**Results:**

Thirty-four fetuses with conotruncal-CHD (22 ToF and 12 D-TGA) and 36 controls were included. ToF-fetuses showed markedly increased cord blood TGFβ (24.9 ng/ml (15.6–45.3) vs. normal heart 15.7 ng/ml (7.2–24.3) vs. D-TGA 12.6 ng/ml (8.7–37.9); *P* = 0.012). These results remained statistically significant even after adjusting for maternal body mass index, birth weight and mode of delivery. TGFß levels showed a negative correlation with the pulmonary valve diameter *z*-score at fetal echocardiography (*r* = −0.576, *P* = 0.039). No other differences were found in the rest of cord blood biomarkers among the study populations. Likewise, no other significant correlations were identified between cardiovascular biomarkers, fetal echocardiography and perinatal outcome.

**Conclusions:**

This study newly describes increased cord blood TGFβ concentrations in ToF compared to D-TGA and normal fetuses. We also demonstrate that TGFβ levels correlate with the severity of right ventricle outflow obstruction. These novel findings open a window of research opportunities on new prognostic and potential preventive strategies.

## 1. Introduction

Conotruncal anomalies are a common group of congenital heart defects (CHD) involving the outflow tracts and great vessels. The inclusion of the outflow tracts views into fetal heart screening ultrasound (1) has greatly improved the prenatal detection of conotruncal anomalies, particularly tetralogy of Fallot (ToF) and D-transposition of great arteries (D-TGA), the two most commonly prenatally diagnosed cyanotic CHD (2). Multidisciplinary care from fetal life has also contributed to greatly increase neonatal survival (3). Therefore, fetal cardiology is now focused on improving medium- and long-term prognostic evaluation.

Several blood cardiovascular biomarkers have been proposed as potential prognostic factors. Previous reports suggest altered circulating concentrations of angiogenic factors in fetuses with CHD. Placental growth factor (PlGF) is a glycoprotein mainly produced in placental trophoblast to promote endothelial growth but also expressed by cardiomyocytes in response to stress (4). The soluble form of fms-like tyrosine kinase-1 (sFlt-1) is a potent antagonist of PlGF that prevents its interaction with cell receptors (5). However, only a few studies have evaluated the pattern of these biomarkers in fetuses with CHD showing controversial results. An antiangiogenic imbalance, with significantly increased cord blood sFlt-1 levels, was firstly described in a mixed group of CHD (6). Recently, a proangiogenic profile with drastically reduced cord blood sFlt1 concentrations, has been reported in a group of left univentricular CHD including hypoplastic left heart syndrome, severe aortic stenosis and Shone syndrome (7). Nonetheless, the role of PlGF and sFlt1 in different types of CHD and their correlation with cardiac dysfunction and perinatal outcome has been insufficiently studied to date.

Additional biomarkers with a potential role in CHD are B-type natriuretic peptide (BNP) and its N-terminal precursor (NT-proBNP). They are released from ventricular myocytes in response to pressure/volume overload or hypoxia, and are clinically useful for CHD screening in neonatal stage (8). Moreover, increased cord blood levels of NT-proBNP and Troponin I, a specific marker of myocardial damage (9), have been described in fetuses with single ventricle (7, 10). Nonetheless, elevated cord blood levels of NT-proBNP and Troponin I have also been reported in neonates with acidosis (11) and intrauterine growth restriction (12).

Lastly, transforming growth factor β1 (TGFβ) is a cytokine produced by different cells with an essential role in the development of heart remodeling and fibrosis (13). Several studies describe elevated plasmatic concentrations of TGFβ in adolescents and young adults with repaired ToF, indicating altered TGFβ signaling in ToF correlating with aortic root dilation (14, 15). To our knowledge, only one study has evaluated this biomarker in fetal life, demonstrating a significant elevation in aortic coarctation, aortic stenosis and Shone syndrome with biventricular outcome (7).

Thus, the study of these biomarkers in a series specifically composed of common conotruncal anomalies could be of interest to preliminary define its cardiac biomarker profile from fetal life, evaluating its potential clinical applicability in subsequent studies. We aimed to first describe the cord blood levels of PlGF, sFlt1, BNP, Troponin I and TGFβ in a prospective series of fetuses with ToF and D-TGA and to explore their correlation with fetal echocardiography and perinatal outcome.

## 2. Materials and methods

### 2.1. Study population

A prospective cohort study was conducted between January 2014 and December 2019, including fetuses diagnosed with ToF and D-TGA at the Fetal Cardiology Unit of BCNatal, which groups two tertiary referral centers for CHD in Barcelona (Clínic and Sant Joan de Déu hospitals). Fetuses with structurally normal hearts were also recruited from low-risk pregnancies attended at BCNatal and included as a control group. Pregnancies of women older than 18 years with accurate gestational age (GA) calculated by first-trimester crown-rump length (16) were considered eligible. Fetal ultrasound and echocardiography were performed in the third trimester, cord blood was obtained at delivery and perinatal results and cardiovascular outcome data were collected postnatally. The study was approved by the institutional Ethics Committee (Reg. HCB/2019/0540). Written consent was obtained from all pregnant women.

ToF was defined by the combination of a subaortic ventricular septal defect with an overriding aorta and infundibular pulmonary obstruction. ToF-cases were sub-classified as ToF with pulmonary stenosis or atresia, based on the presence or absence of anterograde flow through the pulmonary valve, respectively. To obtain a homogeneous group, infrequent cases of TOF with absent pulmonary valve or with major aortopulmonary collaterals were not considered eligible for the study. D-TGA was defined based on the presence a discordant ventricular arterial connection and it was later subdivided into two categories: simple-D-TGA and complex-D-TGA (in the presence of a ventricular septal defect, pulmonary stenosis and/or coarctation of the aorta). All fetuses underwent prenatal genetic testing with microarray analysis and complete extracardiac anatomical ultrasound at diagnosis and during follow-up. Fetuses with pre or postnatal diagnosis of additional major cardiac defects, major extracardiac malformations and/or chromosomal abnormalities were excluded from the study.

Control fetuses were recruited from singleton, spontaneously conceived and low-risk pregnancies attended at the maternal-fetal Medicine Department at BCNatal. Control fetuses were matched for GA (±2 weeks) at delivery with conotruncal-CHD fetuses. Exclusion criteria for controls were pre or postnatal diagnosis of CHD, major extracardiac malformations, chromosomal abnormalities and/or those conditions potentially affecting cord blood biomarkers such as intrauterine growth restriction (12, 17–19), macrosomia (20), pregestational diabetes (20, 21), pregestational hypertension or exposure to toxics (22).

### 2.2. Baseline, perinatal characteristics and cardiovascular outcome

Maternal age, body mass index, ethnicity, smoking status, pre-gestational medical conditions and parity were collected from medical records. Pregnancy outcome, including the presence of intrauterine growth restriction, preeclampsia, pregnancy induced hypertension, gestational diabetes and prematurity below 37 weeks and perinatal characteristics as GA at delivery, mode of delivery, birthweight, neonatal height and head circumference, umbilical artery pH and Apgar score were also recorded. Intrauterine growth restriction was defined as EFW and birthweight below the 3rd centile or below the 10th centile with abnormal uterine, umbilical or middle cerebral artery Doppler values (23). In all cases, CHD subtype was confirmed by postnatal echocardiography and clinical outcome was obtained from medical records at least one year after birth and reevaluated yearly if necessary.

### 2.3. Fetal ultrasound and echocardiography

Fetal ultrasound and echocardiography were performed using a Siemens Sonoline Antares (Siemens Medical Systems, Malvern; PA, USA) or Voluson E10 (General Electric, Zipf, Austria) using a curved-array 2–6 MHz transducer. Structural fetal ultrasound encompassed a detailed extra-cardiac and cardiac examination, following recommended guidelines (24, 25).

Third trimester standard obstetric ultrasound comprised estimation of fetal weight, measurement of mean uterine arteries pulsatility index (PI), umbilical artery PI, middle cerebral artery PI, aortic isthmus PI (26) and ductus venosus PI (27). Estimated fetal weight (EFW) was calculated according to the method of Hadlock et al. (28). EFW centile was calculated using institutional reference curves (29). The cerebroplacental ratio was calculated by dividing the middle cerebral artery PI by the umbilical artery PI (30).

Following the echocardiographic protocol of our center in CHD, in fetuses with ToF and D-TGA, a detailed study of cardiac morphometry and functionalism was performed including measurement of cardiac area, cardiothoracic ratio, ventricular widths, lengths, right-to-left and sphericity indices (SI) and septal thickness from an apical or transverse four-chamber view at end-diastole (31, 32). Aortic and pulmonary valve diameters at mid-systole and aortic-to-pulmonary valve ratio were also obtained Aortic and pulmonary valve size were normalized for gestational age and the *z*-scores were calculated (33).Cardiac function evaluation included aortic and pulmonary peak systolic velocities, mitral (MAPSE) and tricuspid annular-plane systolic excursion (TAPSE) (34).

### 2.4. Cord blood biomarkers

Cord blood samples were obtained from the umbilical vein after cord clamping at birth. Plasma was separated from ethylenediaminetetraacetic acid-treated blood using centrifugation at 1,400× g for 10 min at 4°C. Serum was separated using centrifugation at 2,000× g for 10 min at room temperature. Sample aliquots were immediately stored at −80°C until assayed.

Cord blood biomarkers were measured as previously described (7). Briefly, concentrations of PlGF and sFlt1 were determined in serum by the fully automated Elecsys assays on an electrochemiluminescence immunoassay platform Cobas analyzer (Roche Diagnostics, Mannheim, Germany). NT-proBNP and Troponin I concentrations were measured in plasma by electrochemiluminescence immunoassay using Siemens Atellica IM NT-proBNP and High Sensitivity Troponin I (sensitivity of the technique: 0.0025 pg/ml), respectively (Siemens Healthcare, Erlangen, Germany). TGFβ was measured in serum by conventional ELISA assay Quantikine Human TGF-beta1 (R&D Systems, Minneapolis, MN, USA). Concentrations of cord concentrations of PlGF, sFlt1, NT-proBNP and TGFβ are presented as continuous variables. Given the different behavior of troponin I (acute increase in response to ischemia or hypoxia), Troponin I was treated as dichotomous variable: concentrations above 0.0093 pg/ml (which corresponds to the 75th centile of the troponin level among the control group), were considered to be high.

### 2.5. Statistical analysis

IBM SPSS Statistics version 25 statistical package (IBM Corp., Armonk, NY, USA) was used for statistical analysis. Kolmogorov-Smirnov test of normality was performed in all continuous variables. Measurements were expressed as mean ± standard deviation or as median (range) for continuous variables as appropriate, and frequencies with percentages for categorical variables. Differences between study groups were examined using parametric analysis of variance (one-way ANOVA) followed by *post hoc* Bonferroni tests for pairwise comparison for normally distributed variables, Kruskal–Wallis one-way ANOVA followed by post-hoc pairwise comparisons using the Dunn–Bonferroni approach for non-normally distributed variables and *χ*^2^ test for categorical variables.

Baseline variables were analyzed to identify possible confounders including maternal age, maternal body mass index (BMI), nulliparity, smoking, pregestational diabetes, gestational diabetes, preeclampsia, GA at delivery, birth weight, gender and mode of delivery. Potentially confounders factors such as maternal BMI, birth weight and mode of delivery were significantly different between groups, therefore were adjusted in the model. Spearman correlation coefficient to compare associations between biomarkers and the previously described standard obstetric ultrasound and echocardiographic parameters was used in the ToF and D-TGA groups. For all analyses, *P*-values <0.05 were considered statistically significant.

## 3. Results

### 3.1. Study population and obstetric standard ultrasound

From the original cohort of 84 ToF and D-TGA fetuses, 18 pregnant women elected for termination of pregnancy. We excluded from the analysis 6 cases with ToF: 1 case with absent pulmonary valve, 1 case with major aorto-pulmonary collateral arteries (MAPCAs), 1 case with intrauterine fetal demise, 1 monochorionic twin pregnancy, 1 case with a chromosomal abnormality and a last case with an associated multicystic kidney disease. We didn't obtain cord blood at delivery in 23 cases, 1 patient was lost to follow-up and 2 refused to participate. The final study population consisted of 34 fetuses with conotruncal CHD (22 ToF and 12 D-TGA cases) and 36 controls.

**Figure 1:**
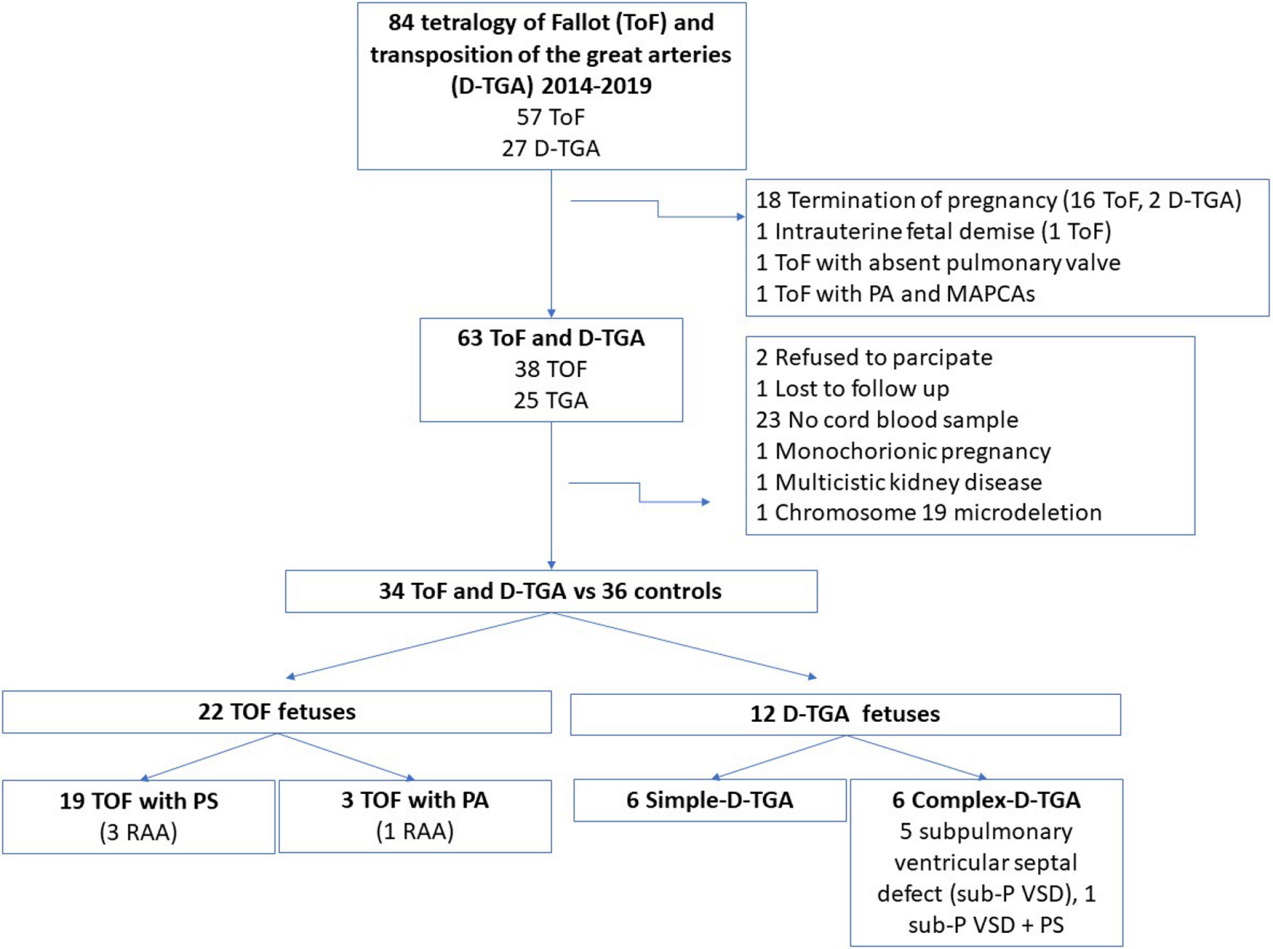
Flow chart of fetuses with conotruncal congenital heart defects included in the study. ToF, tetralogy of fallot, D-TGA, transposition of the great arteries; PA, pulmonary atresia; MAPCAs, major aortopulmonary collateral arteries; RAA, right aortic arch; sub-P VSD, subpulmonary ventricular septal defect; PS, pulmonary stenosis.

As shown in Figure 1, the ToF group included 19 fetuses with pulmonary stenosis and 3 cases with pulmonary atresia. Only one case of ToF with pulmonary stenosis presented retrograde flow at the ductus arteriosus, with the remaining cases presenting forward flow until birth. Blalock-Taussig shunt was required prior to the ToF corrective surgery only in the 3 cases with pulmonary atresia.

The D-TGA-group included 6 fetuses with a simple-D-TGA, 5 cases with a subpulmonary ventricular septal defect and one fetus with an associated pulmonary stenosis (complex-D-TGA group). Rashkind atrioseptostomy was performed prior to arterial switch procedure in 58.3% of the D-TGA fetuses (7/12). The cardiovascular outcome was favorable in all conotruncal CHD cases, with no significant postnatal complications and with a median follow-up of 46 months (interquartile range 28.0–50.5).

Maternal baseline characteristics and perinatal results are shown in Table 1. Most baseline characteristics were similar in the three study populations; however, BMI was significantly higher in pregnant women with ToF-fetuses compared to the other two groups. Additionally, the prevalence of small for GA fetuses was higher in the ToF group [31.8% (7/22)] and cesarean section was more frequently performed in the ToF and D-TGA groups compared to controls. Additional perinatal outcomes such as GA at delivery was similar across groups and there were no differences in the five-minute Apgar score and umbilical artery pH. No cases of fetal acidosis were found. Regarding biometrics at birth, ToF-fetuses had a significantly lower birth weight compared to D-TGA and control groups, with only 2 ToF cases with pulmonary stenosis presenting a birth weight below the 3th percentile. The head circumference at birth was also significantly reduced in both conotruncal CHD groups as compared to controls. As shown in Table 1, birth length did not significantly differ between the three groups.

**Table 1:** Maternal characteristics and perinatal outcome in the study populations.

Variable	Tetralogy of fallot (*n* = 22)	Transposition of the great arteries (*n* = 12)	Controls (*n* = 36)	*P*-value
**MATERNAL CHARACTERISTICS**				
Maternal age (years)	35.27 ± 6.35	33.58 ± 5.14	33.88 ± 4.67	0.560
Body mass index (kg/m^2^)	28.4 (24.29–29.31)*	26.51 (24.24–30.85)	22.92 (21.36–24.39)	**<0** **.** **001**
Chronic diseases	6 (27.27%)	2 (16.67%)	11 (30.6%)	0.576
Ethnicity				0.596
Caucasian	15 (68.2%)	11 (91.7%)	26 (72.2%)	
Latin American	2 (9.1%)	1 (8.3%)	2 (5.6%)	
Maghreb	4 (18.2%)	0 (0%)	3 (8.3%)	
Asian	1 (4.5%)	0 (0%)	4 (11.1%)	
African	0 (%)	0 (%)	1 (2.8%)	
Smoking habit	0 (0%)	1 (8.3%)	1 (2.8%)	0.378
Nulliparity	11 (50%)	8 (66.7%)	17 (47.2%)	0.499
**PERINATAL CHARACTERISTICS**				
Gestational age at birth (weeks)	39.4 (38.2–40.0)	40 (39.5–41.1)	39.5 (39.0–40.2)	0.326
Pregnancy complications:				
* *Small for gestational age	7 (31.8%)*	2 (16.7%)*	0 (0%)	**0.002**
* *Intrauterine growth restriction	2 (9.1%)	1 (8.3%)	0 (0%)	0.189
* *Preeclampsia or pregnancy induced hypertension	0 (0%)	0 (0%)	0 (0%)	1
* *Gestational diabetes	1 (4.5%)	0 (0%)	3 (8.3%)	0.538
* *Prematurity (Birth <37 weeks)	0 (0.0%)	0 (0.0%)	1 (2.8%)	0.619
Cesarean section	12 (54.5%)*	4 (33.3%)*	8 (22.2%)	**0** **.** **042**
Birth weight (g)	2,935 (2,729–3,305)*	3,235 (3,077–3,465)	3,390 (3,132–3,637)	**0** **.** **004**
Birth weight centile	32 (9–59)*	35 (20–64)	43 (38–70)	**0** **.** **007**
Birth weight <3rd centile	2 (9.1%)	0 (0%)	0 (0%)	0.106
Birth weight >4,000 g	0 (0%)	0 (0%)	0 (0%)	1
Five-minute Apgar <7	2 (9.1%)	1 (8.3%)	2 (5.6%)	0.866
Umbilical artery pH	7.25 (7.20–7.28)	7.24 (7.2–7.29)	7.21 (7.16–7.28)	0.164
Female fetal gender	4 (18.2%)	6 (50%)	16 (44.4%)	0.080
Head circumference at birth (cm)	33.6 (32.3–38.8)*	33.7 (32.1–34.4)*	35.4 (34.5–36.0)	**<0** **.** **001**
Head circumference centile	34 (13.5–80.7)	21 (4.25–39.75)*	72 (52.75–84.25)	**<0** **.** **001**
Height (cm)	50 (47–51.25)	50.5 (49–52.5)	51 (50–52)	0.125
Height centile	34 (13.5–80.75)	59.5 (31.75–89.25)	70.5 (40.5–89.8)	0.066
**CARDIOVASCULAR OUTCOME**				
Follow-up (months)	47 (33.5–51.5)	35.5 (6.5–50.7)	NA	

Data expressed as mean ± standard deviation, median (interquartile range) or *n* (percentage). ToF, tetralogy of fallot; PS, pulmonary stenosis; D-TGA, transposition of the great arteries; NA, non-applicable.

*P*-value calculated by ANOVA.

^Ψ^ Bold numbers means statistically significant.

* Denotes *P* < 0.05 as compared to controls (Post-hoc comparisons using Dunn–Bonferroni for continuous and *χ*^2^ for categorical variables).

Table 2 details the results of fetal ultrasound in the study populations. Interestingly, there were no significant differences in estimated fetal weight or head circumference at the time of ultrasound. There were also no differences in uterine or umbilical-fetal and ductus venosus Doppler between the three groups.

**Table 2:** Feto-placental ultrasound in the study populations.

Variable	Tetralogy of Fallot (*n* = 22)	Transposition of the great arteries (*n* = 12)	Controls (*n* = 36)	*P*-value
Gestational age at ultrasound (weeks)	33.3 (31.5–33.2)	34.1 (31.1–34.6)	33.1 (29–35.1)	0.193
Estimated fetal weight (g)	2,033.48 ± 405.06	2,144.08 ± 579.13	1,908.72 ± 694.40	0.464
Estimated fetal weight centile	28 (13–61)	51 (30–83)	43 (17–62)	0.221
Head circumference (mm)	304 (292–319)	294 (272–313)	295 (255–312)	0.271
Mean uterine artery PI centile	56.85 ± 28.67	49.17 ± 17.48	35.81 ± 31.98	0.098
Mean uterine artery PI > p95	2 (15.4%)	0 (0%)	2 (6.3%)	0.415
Umbilical artery PI centile	51.76 ± 24.10	39.0 ± 21.70	44.34 ± 23.31	0.302
Umbilical artery PI < p95	0 (0%)	0 (0%)	0 (0%)	1
Middle cerebral artery PI centile	52 (32.5–74)	39 (18–95)	51 (21–71)	0.785
Middle cerebral artery PI < P5	0 (0%)	0 (0%)	1 (2.9%)	0.629
Cerebroplacental ratio	2.05 ± 0.67	2.43 ± 1.03	2.04 ± 0.52	0.216
Cerebroplacental ratio centile	40.10 ± 26.87	51.55 ± 37.86	47.49 ± 30.63	0.561
Cerebroplacental ratio < P5	0 (0%)	0 (0%)	2 (5.7%)	0.401
Ductus venosus PI centile	52.18 ± 29.65	46.70 ± 30.78	36.18 ± 31.46	0.207
Aortic isthmus PI centile	58.36 ± 12.70	65.86 ± 20.09	71.41 ± 14.41	0.056

Data expressed as mean ± standard deviation, median (interquartile range) or *n* (percentage). PI, pulsatility index.

*P*-value calculated by ANOVA.

### 3.2. Cord blood biomarkers

**Figure 2:**
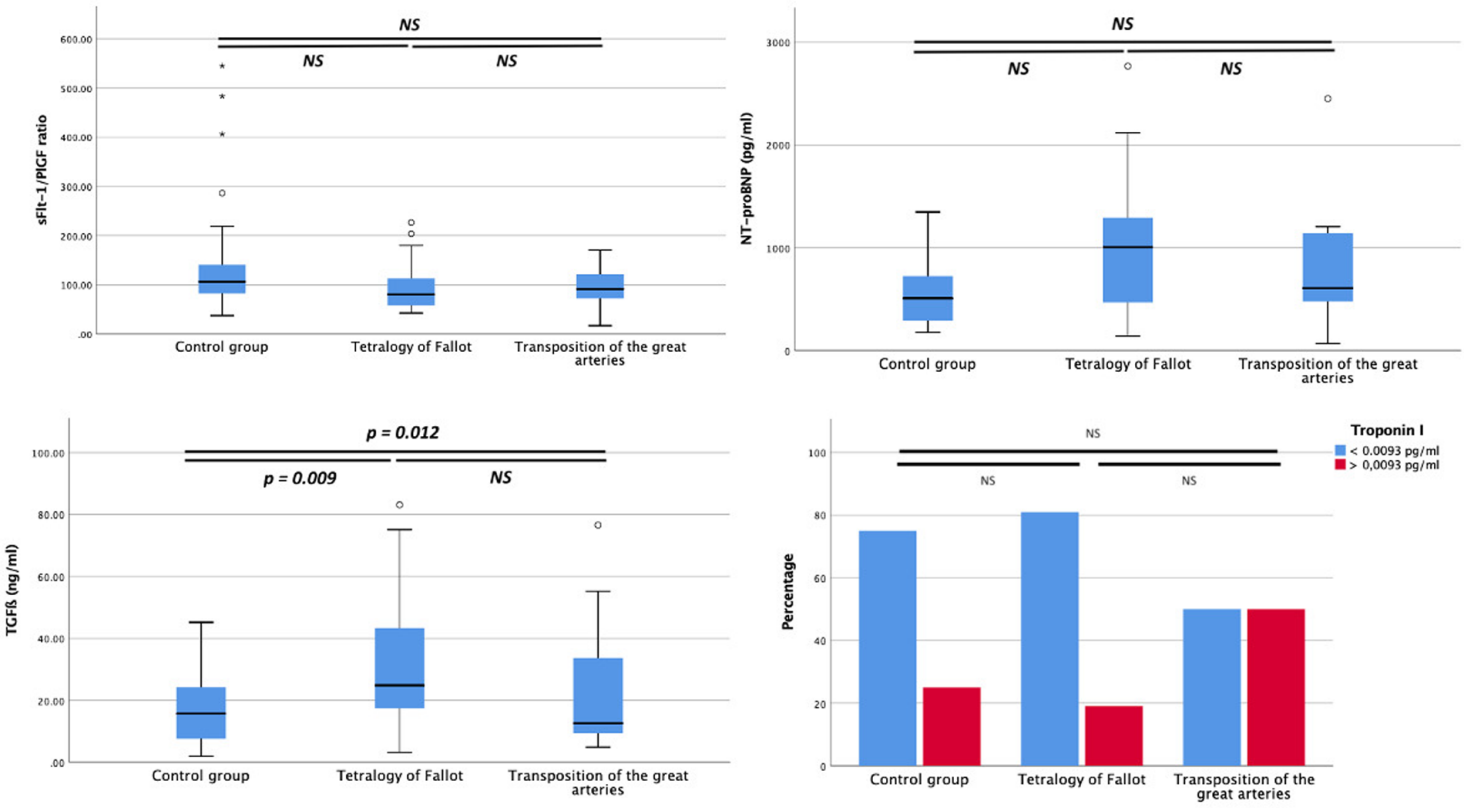
Umbilical cord concentrations of tyrosine kinase-1/placental growth factor (sFlt-1/PlGF) ratio, N-terminal precursor of B-type natriuretic peptide, transforming growth factor ß (TGFß) and percentage of fetuses with troponin I positive (considered positive if concentration >0.0093 pg/ml which represents 75th centile in the control population), in fetuses with tetralogy of fallot, transposition of the great arteries and in normal fetuses. *P < *0.05 is considered statistically significant. NS: not significant.

Results of cord blood biomarkers in ToF, D-TGA and control-groups are presented in Table 3 and Figure 2. Concentrations of cord blood angiogenic factors including PlGF, sFlt1 and sFlt1/PlGF ratio, did not show significant differences among the study populations. Compared with the controls, ToF fetuses presented a marked increase in cord blood concentrations of TGFβ. Troponin showed a non-significant tendency to higher concentrations in the D-TGA group (Figure 2).

**Table 3:** Concentrations of biomarkers in cord blood the study populations.

Variable	Tetralogy of fallot (*n* = 22)	Transposition of the great arteries (*n* = 12)	Controls (*n* = 36)	Adjusted *P*-value
Placental growth factor (PlGF) (pg/ml)	12.7 (10.9–20.2)	14.6 (11.15–29.55)	17.5 (13.7–23.55)	0.222
Soluble fms-like tyrosin kinase 1 (sFlt-1) (pg/ml)	1,116.6 (856.9–1,966.2)	1,121.2 (912.75–1,946.9)	1,962.3 (1,379.9–2,726.25)	0.711
sFlt-1/PlGF ratio	80.36 (57.76–115.73)	91.46 (63.89–125.03)	106.25 (78.48–155.22)	0.401
High troponin I^Ψ^ positive	5 (19%)	6 (50%)	8 (25%)	
Pro-Brain natriuretic peptide (pg/ml)	1,005 (466.5–1,588)	607 (471.75–1,157.75)	508 (288.5–740.75)	0.154
Transforming growth factor β (ng/ml)	24.85 (15.75–45.28)*	12.6 (8.7–37.9)	15.7 (7.25–24.3)	**0** **.** **012**

Data expressed as mean ± standard deviation, median (interquartile range) or *n* (percentage).

^Ψ^ Troponin considered positive if concentration >0.0093 pg/ml which represents 75th centile in the control population.

*P*-value calculated by logistic regression adjusted by body mass index, cesarean section and birthweight.

Bold numbers means statistically significant.

^*^ Denotes P<0.05 between Tetralogy of Fallot and Controls (post-hoc comparisons using Dunn-Bonferroni for continuous variables).

Cord blood biomarkers were not correlated with any obstetric ultrasound parameter, such us umbilical artery, middle cerebral artery and aortic isthmus PI, nor with any perinatal outcome including mode of delivery, estimated fetal weight, head circumference, umbilical artery pH and 5 min Apgar score.

**Figure 3:**
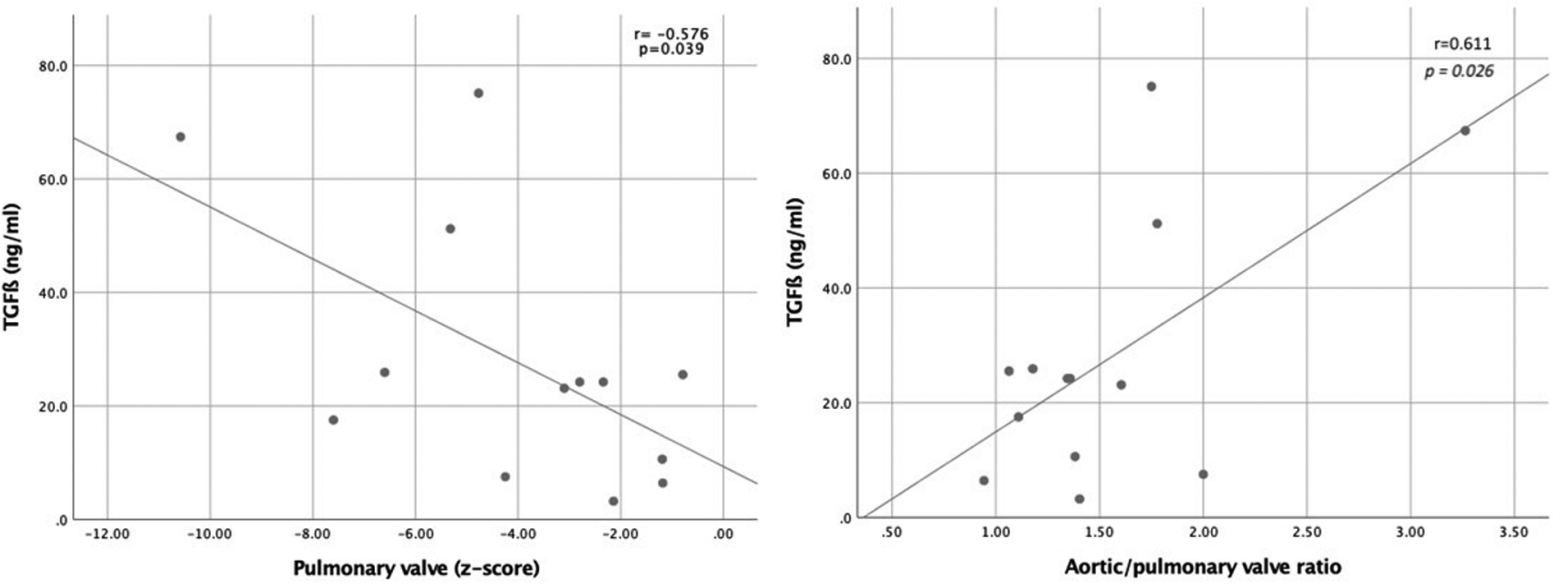
Relationship between umbilical cord concentration of transforming growth factor ß (TGFß) and pulmonary valve diameter *z*-scoress (left graphic) and aorta/pulmonary ratio (right graphic).

Finally, and as shown in Figure 3, cord blood levels of TGFβ showed a negative correlation with the pulmonary valve diameter *z*-score (*r* = −0.576, *P* = 0.039) and positive correlation with aortic/pulmonary valve ratio (*r* = 0.611, *P* = 0.026) in ToF fetuses. No additional correlations were found between cord blood biomarkers and the rest of morphometric and functional echocardiographic parameters evaluated in ToF and D-TGA fetuses, including aortic peak systolic velocity.

## 4. Discussion

This study first describes the pattern of different cord blood biomarkers in a cohort of fetuses diagnosed with isolated ToF and D-TGA. Our main findings are: (1) TGFβ is significantly increased in ToF from fetal stage and (2) cord blood TGFβ levels correlate with the severity of the prenatal right ventricular outflow tract obstruction. Only a few prior studies have evaluated cord blood cardiovascular biomarkers in fetuses with CHD with conflicting results. Differences in the CHD groups included among the studies, together with their small sample size, precludes direct comparison of results. A series of 39 fetuses with a mixed group of CHD, which included some cases with conotruncal anomalies reported an anti-angiogenic pattern with increased cord blood sFlt-1 levels (6). On the contrary, a prior study by our group, evaluating 45 fetuses with left-CHD, found only modestly decreased PlGF in the left-CHD group compared to normal fetuses, and markedly decreased sFlt1 only in fetuses with univentricular left-CHD conferring a proangiogenic profile of PlGF and SFlt1 in the poor prognostic group (7). In our study, no differences were found in cord blood levels of PlGF and sFlt1 among the study groups. Given that sFlt1 is downregulated by hypoxia (35), our results in conotruncal CHD suggest that there is little ventricular hypoxic damage *in utero* in these cyanotic CHD. Further studies are needed to better define which groups of CHD are related to abnormal angiogenesis at the cardiac level and its possible relationship with a concomitant deficient placental angiogenesis (36).

Several studies have reported cord blood NT-proBNP to be elevated in CHD, including 10 cases with mixed CHD (12), 15 fetuses with functional single ventricle associated to neonatal death (10), 16 fetuses with univentricular left-CHD (7) and 6 cases with non-immune hydrops of cardiac origin (10). Although these studies group a limited number of cases, the results are consistent with prior data reporting elevated NT-proBNP in pediatric patients (37) with heart failure, indicating that NT-proBNP is a useful predictor in the setting of severe cardiac anomalies. In our study, NT-proBNP was slightly elevated in the ToF group and preserved in D-TGA cases, which is in accordance with the favorable biventricular outcome and lack of major postnatal complications of our series.

Finally, there is very limited data on Troponin I behavior in fetuses with CHD. We previously reported positive cord blood Troponin I to be more frequent in the group of fetuses with left-CHD with favorable cardiac outcome (7). In the present study, a higher proportion of fetuses with D-TGA presented positive values above the 75th centile (Figure 2), but this difference was not significant possibly due to the limited number of cases. Troponin I is a very sensitive biomarker for endocardial hypoxia and myocardial damage. It has also been found to be elevated in newborns requiring NICU admission (38), in neonates with acidemia (39) and in fetuses with intrauterine growth restriction (40). Thus, larger populations studies are needed to better describe the profile of all these biomarkers not only in different groups of CHD but also in clinical conditions associated with cardiac dysfunction.

### 4.1. Increased cord blood TGFß in ToF compared with D-TGA

This is the first prenatal report of increased cord blood TGFβ concentrations in ToF. This increase may be explained by different mechanisms. First, TGFβ has been identified as a primary factor responsible for cardiovascular fibrosis (41) and upregulation of TGFβ signaling has been shown to be associated with pro-fibrotic molecular signaling in cardiac pressure overload (42). In this regard, limited data is available on blood flow mechanics and its effect on biventricular remodeling in fetal ToF (43). Nonetheless, a recent computational model evaluating fluid dynamics in fetuses with ToF has shown that although biventricular pressure is equalized by the presence of the ventricular septal defect, biventricular pressure is globally elevated in ToF compared to normal hearts leading to mild right ventricle hypertrophy in fetal life (44). These findings could plausibly explain the higher levels of TGFβ in the ToF group. Furthermore, the ventricular walls around the ventricular septal defect consistently experienced high stress due to a shear flow effect, a mechanism which has also been described to induce cardiovascular tissue growth and remodeling (45).

Secondly, in normal fetal circulation there is a preferential shunting across the foramen ovale that comprises 30% of the cardiac output (46). However, tricuspid flow has been described to be increased in ToF (44), as a lower resistance alternative to the foramen ovale. As such, some of the right atrial flow is derived through the right ventricle and the ventricular septal defect instead of flowing through the foramen ovale, further contributing to the increased endothelial shear stress at the ventricular septal defect.

Finally, TGFβ has also a central role in vascular morphogenesis and extracellular matrix homeostasis, thus there is a growing interest to better understand its contribution to vascular remodeling. Altered TGFβ signaling has been reported in bicuspid aortopathy as a key component in the pathogenesis of thoracic aneurysms (47) as well as in Marfan syndrome, in which circulating levels of TGFβ are correlated with aortic root dilation (48). Moreover, overexpression of TGFβ in the ascending aorta has been described in patients with ToF, tricuspid atresia and double-outlet right ventricle in association with abnormal elastic fibers. Interestingly, increased TGFβ levels have been recently found in a study evaluating adolescents and young adults with TOF after surgery in comparison with controls (14). TGFβ was also slightly elevated after atrial switch operation in corrected TGA and Fontan procedures but was preserved after arterial switch surgery in D-TGA. These data suggest that different mechanisms may be involved in the neo-aortic root dilation that progressively occurs in repaired D-TGA compared to other CHD. Our data, showing similar results in fetuses with ToF and D-TGA, reinforce the hypothesis that an underlying lesion of the aorta may already be present in ToF from very early stages of development. In fact, a decreased aortic compliance, evaluated by fetal echocardiography, has already been reported in fetuses with Marfan syndrome and ToF compared to normal fetuses (49). Therefore, future studies evaluating fetal aortic characteristics in different CHD are warrantied.

### 4.2. TGFß correlates with the severity of the right ventricle obstruction in ToF

Prior studies have demonstrated a positive correlation between circulating levels of TGFβ and aortic sinus dimension in patients with repaired-ToF (14). Moreover, TGFβ levels have also been correlated with the aortic stiffness evaluated by echocardiography in patients with ToF before the surgical repair (15). Our data are consistent with these findings. As shown in Figure 3, TGFβ concentration showed a negative correlation with the pulmonary valve diameter and a positive correlation with aortic/pulmonary valve ratio in ToF fetuses. Thus, we could hypothesize that increasing right ventricular obstruction severity results in progressive aortic volume overload, which may be associated with a more pronounced deleterious hemodynamic effect and higher TGFβ levels. However, we could not demonstrate any correlation between TGFβ levels and fetal right ventricular morphometry and functionalism, assessed by the sphericity index and tricuspid annular-plane systolic excursion, respectively; nor with peak pulmonary/aortic systolic velocity, findings that require to be conformation with a larger number of cases.

In agreement with previous reports, ToF-newborns had a significantly lower birth weight compared to D-TGA and control groups (50). Additionally, head circumference perimeter at birth was also significantly reduced in both conotruncal CHD groups (51). However, cord blood biomarkers were neither correlated with any ultrasound parameter and perinatal outcome. Likewise, no cord blood biomarker was correlated with any echocardiographic parameter in the D-TGA and control groups. However, these results may be limited by the small sample size of our study.

### 4.3. Strengths and limitations

To our knowledge, this is the first study to perform a comprehensive assessment of cord blood cardiac biomarkers profile in a specific series of fetuses diagnosed with conotruncal anomalies. It is also the first study to identify increased cord blood TGFβ concentrations in ToF and its correlation with the severity of right ventricular outflow tract obstruction.

However, this is an exploratory study and, therefore, future studies are necessary to confirm our findings and subsequently evaluate the potential clinical applicability of TGFβ in the prognostic evaluation of ToF. The main limitation of our study is the limited number of cases, especially in the D-TGA group. However, the group of ToF is quite homogeneous since most of the cases correspond to ToF with pulmonary stenosis. Although cord blood biomarkers concentrations were analyzed after adjustment for identified confounders we recognize that additional confounders might exist. Finally, we are aware that we have neither the weight of the placenta at birth nor a placental biopsy, which could also provide relevant information.

## 5. Conclusions

This study newly describes increased cord blood TGFβ concentrations in ToF compared to D-TGA and normal fetuses. Moreover, we demonstrate that TGFβ levels correlate with the severity of right ventricle outflow obstruction. These novel findings open a window of research opportunities into new prognostic and potential preventive strategies. For this, larger multicenter studies, including enough cases in each category of conotruncal anomalies and other groups of CHD, both in fetal and postnatal stages, are warranted. Additionally, the study of these cardiovascular biomarkers in maternal blood and as well as in the amniotic fluid would allow to expand their potential clinical applicability to earlier stages of gestation.

## Data Availability

The raw data supporting the conclusions of this article will be made available by the authors, without undue reservation.
